# The Impact of Climate Change on the Urban Tree *Ailanthus altissima*: Insights from More than Four Decades of Pollen Data in Vienna (Austria)

**DOI:** 10.3390/plants14243823

**Published:** 2025-12-16

**Authors:** Maximilian Bastl, Katharina Bastl, Karen Koelzer, Marija Aleksic, Christina Morgenstern, Martin Schepelmann

**Affiliations:** 1 Department of Otorhinolaryngology, Medical University of Vienna, Währinger Gürtel 18–20, 1090 Vienna, Austria; 2 Division of Structural and Functional Botany, Department of Botany and Biodiversity Research, University of Vienna, Rennweg 14, 1030 Vienna, Austria; 3 Institute of Pathophysiology and Allergy Research, Medical University of Vienna, Währinger Gürtel 18–20, 1090 Vienna, Austria

**Keywords:** neophyte, urban environment, tree pollination, flowering period, pollen allergy, temporal trend, aerobiology, palynology, biomonitoring

## Abstract

*Ailanthus altissima* (tree of heaven) is among the most abundant and widespread neophytic plants in Austria. The pollen season of *Ailanthus* usually ranges from the beginning of June until the mid of July, showing one peak period around the mid of June in Vienna (Austria). Over a span of 48 years (1976–2023), pollen data of *Ailanthus* was gathered from aerobiological samples and assessed for a temporal trend. In addition, weather data from Vienna (temperature, precipitation, relative humidity and sun hours) was incorporated to find possible associations with *Ailanthus* pollen indices. The change in the pollen season for *Ailanthus* described in this study has already manifested and is ongoing. Temperature and sunshine hours have a direct impact on the flowering of urban trees, indicating that global climate change may be a major driver towards more intense pollen and allergy seasons.

## 1. Introduction

*Ailanthus altissima* (Mill.) Swingle, belongs to the family Simaroubaceae and is also known as tree of heaven, stinktree, stinking ash or Chinese sumac. It originates from China and North Vietnam but can nowadays be found in all temperate and subtropical regions on all inhabited continents [1,2]. If not further specified, we refer to it as *Ailanthus* herein and state the full species name when referring to a different species. *Ailanthus* is considered in Europe as non-native and invasive and has been declared as one of the hundred most problematic invasive species in Europe [3].

Due to its invasive nature, the tree was added to the list of invasive alien species of Union concern on 25 July 2019, in the EU regulation 2019/1262. This includes, among several other actions, a ban on trade of the tree within the European Union. According to the Federal Environmental Agency of Austria, it is defined as a non-native, invasive plant that poses a high conservation threat [4]. *Ailanthus* has been used as a city and garden tree in the past. It was imported from China to Europe already in the time of 1740–1750 and was, e.g., planted since 1780 as a decorative tree in Berlin, Germany. The sudden large-scale spread occurred 170 years later, after World War II [4,5]. Botanical gardens, the use as a decorative plant, transport by vehicles, and contamination of soil and machines were possible pathways of its spread according to the action plan of invasive alien species in Austria of the Federal Environmental Agency [6].

The robustness of the plant itself is well known, making it a perfect tree in urban environments, where several stress factors are present. *Ailanthus* copes with (i) air pollution [7], (ii) high salinity [8], (iii) drought [9,10] and (iv) problematic soils such as hard-packed [11], infertile [12] and contaminated [13] soils. Furthermore, it outcompetes other plants by containing phytotoxic compounds in its stems, roots and leaves that lead to an allelopathic effect on other plants, especially their seed germination and seedling growth [14,15,16,17]. *Ailanthus* is known as a thermophilic species [1] with the main area of distribution in the warmest part of cities [5] and is strongly associated in Central Europe with urban areas, related land use and the effects of urban heat island [18].

The flowering biology of *Ailanthus* is peculiar as there are male and female trees as well as hermaphroditic trees. The tree is mainly insect-pollinated [19]; however, a certain amount of the pollen becomes airborne, which indicates the ambophilous nature of the tree [18]. Male flowers emit an odor to attract pollinators, which is unpleasant for humans—thus the name stinktree. The pollen production was assessed as 5539 pollen per anther by [18]. However, they note that pollen production varies between individual trees and that the year under study was a year of lower pollen production for *Ailanthus* in general. In comparison, the species *Ailanthus excelsa* shows an exceptionally high pollen production with 327,036 pollen per anther [20]. Overall, relevant pollination from *Ailanthus* is documented despite the insect-pollinated nature of the flowers, as the most recent study from Berlin shows [18].

Furthermore, *Ailanthus* pollen is considered as a relevant allergen [21] after the identification of well-known allergenic proteins in its pollen [22] and the observation of sensitizations and allergic symptoms in atopic patients [23,24,25,26,27]. This is not very surprising, since *Ailanthus* is an important aeroallergen in its native region, China, with sensitization rates of nearly 30% [27].

**Figure 1:**
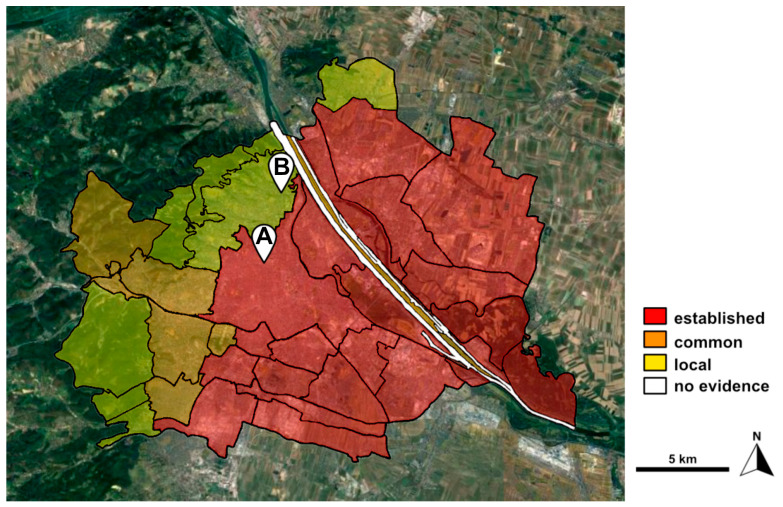
Distribution map of *Ailanthus altissima* in Vienna, adapted from the neobiota strategy and management plan of the Vienna municipal administration with the permission of the original authors [29]. The distribution zones are divided into the following categories: no evidence (white), local (yellow), common (orange) and established (red). The markers show the location of the monitoring station from 1976 to 2002 (A) and from 2003 to 2023 (B).

The situation in Austria and especially the metropolitan area of Vienna is similar to that in other parts of Europe, as well as North America, where it is one of the most widespread invasive alien plant species [28]. In cities, it can cause damage on infrastructure and archeological remains with its roots [28]. *Ailanthus* invades the shrub and tree layer in forests, dry grasslands and neglected grasslands in the Pannonian region of Eastern Austria, which also has a considerable impact on forestry, according to the Federal Environmental Agency of Austria [4]. As presented in the Neobiota strategy and management plan of the Vienna municipal administration, it is established in the Northeast and the Southeast of Vienna as well as in other parts of the city (Figure 1; [29]). Currently, the northern and western parts of Vienna in the vicinity of the Vienna Woods and the area around the Bisamberg show only a rare occurrence (Figure 1; [29]). Within the built-up, public areas of Vienna, all trees are managed by the municipality and can be looked up in the open-access online tree register of the municipal authorities of the city of Vienna [30]. Surprisingly, out of almost 100,000 trees, there were only 416 *Ailanthus altissima* trees officially registered in the year 2024, as presented in the statistical yearbook of the city of Vienna [31]. These numbers, however, only include trees planted for ornamental reasons. They do not include spontaneous vegetation on brownfields, rail tracts or privately owned land. Hence, most *Ailanthus* trees in Vienna are unregistered and spread in an unmonitored way, which makes it difficult to assess the dispersion of the plant in the study area in detail. The municipal authorities of Vienna are occupied with the control of *Ailanthus* and prevent a further spread or even remove this tree from conservation areas, nature reserves, protected biotopes/habitats or European nature reserves, as mentioned in the neobiota strategy and management plan [29]. The same reference reports that 8500 trees have already been removed in Vienna from 2015 up to now. However, it is stated that measures follow the cost/benefit principle. This means that a large-scale control measure is not indicated in areas where *Ailanthus* has already successfully established, because costs would be too high due to the longevity of the seed reservoir (seeds may germinate after ten years; [29]).

Management methods were recently reviewed [32] and comprise mechanical, chemical and biological control. A combination of mechanical removal and chemical control of the cut stump seemed to be the best procedure. Further challenges, such as soil/water contamination due to herbicides, vegetal residues and their allelopathic compounds, and potential effects of the biological control method with the fungus *Verticillum* [32] must be addressed.

Summarizing, many aspects on the biology and management of *Ailanthus* as described above are well documented. It is thus surprising that data on *Ailanthus* pollen concentrations are rarely published and there is especially no knowledge on the development of pollen concentrations through the recent years or decades. The monitoring of an invasive neophyte that has allergenic potential should be included in pollen monitoring activities as well.

This study is the first to close this gap and analyzes the data series of more than 40 years of monitoring airborne *Ailanthus* pollen in Vienna (Austria). By tracking seasonal pollen concentrations over time, variations in pollination intensities will be assessed and linked to a climate change-induced shift in distribution patterns. To the best of our knowledge, this is the longest time series of *Ailanthus* pollen data published so far.

## 2. Results

### 2.1. Ailanthus Pollen Season from 1976 to 2023 in Vienna

The historical *Ailanthus altissima* pollen dataset (1976–2023) underwent a quality control assessment to evaluate data reliability (see Material and Methods).

The pollen seasons of *Ailanthus altissima* in Vienna exhibit relatively stable timing, with mean start and end dates on day 158 (June 7 ± 11 days) and day 194 (July 13 ± 16 days) of the year, respectively. The average season duration spans 37 days (±17 days), producing an annual pollen concentration of 82 grains/m^3^ (±41 grains/m^3^). Peak pollen levels occur around day 167 (June 16 ± 10 days), followed by distinct pre- and post-peak phases lasting 11 (±7 days) and 27 days (±16 days), respectively. These phases show marked variability in pollen integrals (44 ± 28 grains/m^3^ pre-peak; 38 ± 23 grains/m^3^ post-peak) (Table 1). For individual pollen season descriptors, see Supplementary Table S3.

**Figure 2:**
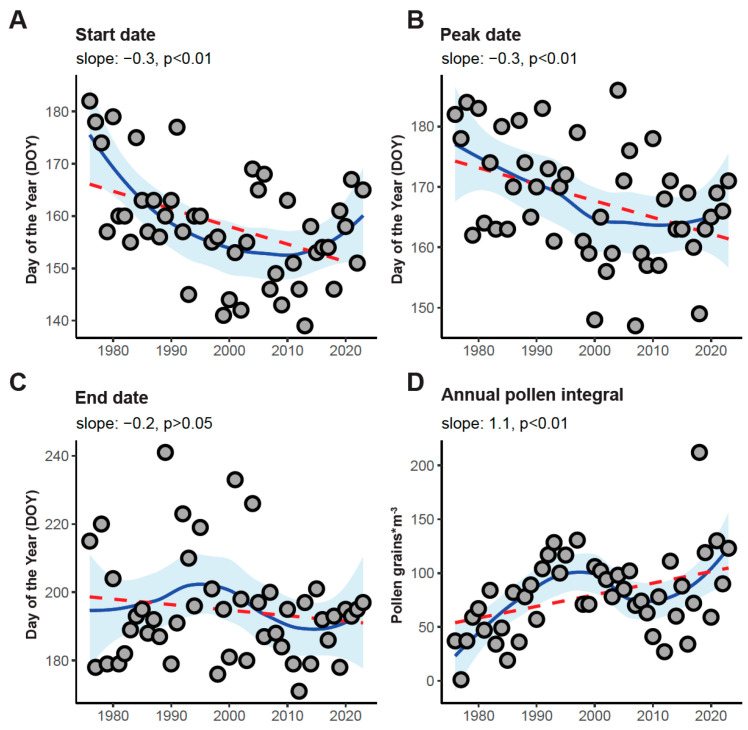
Trend analysis of historic *Ailanthus altissima* pollen data (1976–2023, excluding 1996). Linear regression for start (**A**), peak (**B**), end (**C**) and annual pollen integral (**D**). Filled circles denote individual data points. Blue lines show the smoothed trend lines with 95% confidence intervals shaded in light blue. Red dashed lines represent fitted linear regression lines.

To evaluate temporal trends in the parameters of the pollen season, linear regression analyses were conducted. Significant trends were identified for season onset, peak timing, and annual pollen integrals, highlighting shifts in phenological and aerobiological patterns over time (Figure 2; Table 2). While start and peak dates were shifting towards earlier time points of the year, the end date remained stable across the time period.

**Figure 3:**
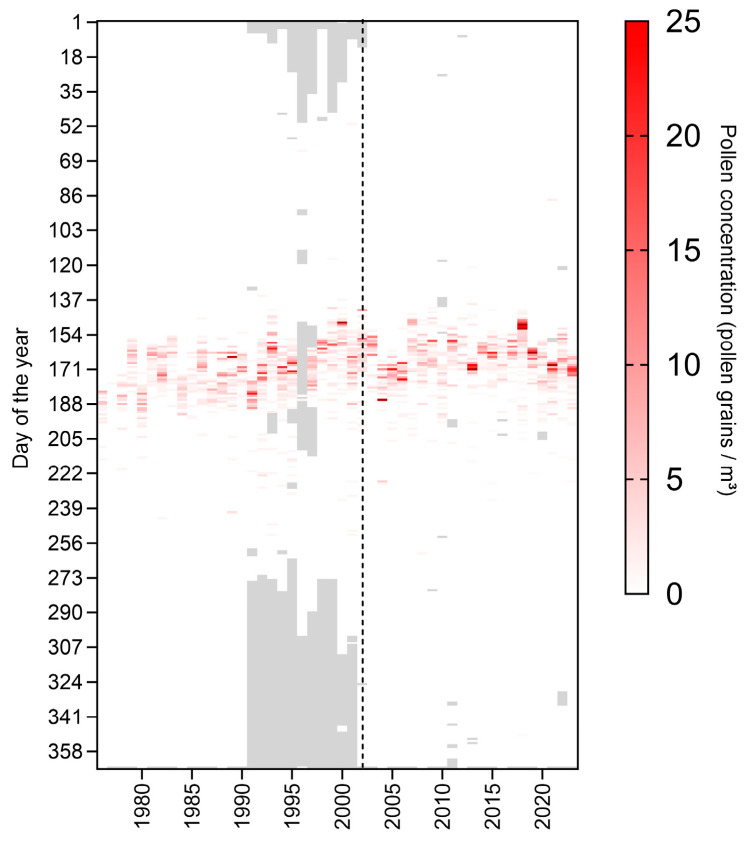
Heatmap of the daily pollen concentration (grains per m^−3^) of *Ailanthus* at the General Hospital of Vienna (AKH) for the years 1976–2002 and at Geosphere Austria, Vienna, 2003–2023. Gray areas indicate missing values. White indicates 0 (no pollen). The dotted line indicates the year 2002/2003 where the pollen collection point was moved between the two sites. The year 1996 was excluded from all further analysis due to a high number of missing values during the pollen season.

When comparing the pollen concentrations throughout the day of the years of the whole time period, it becomes apparent that pollen concentrations increase first in the 90s of the last decade and a second time from 2010 onwards (Figure 3). The change in the monitoring site for 2003, indicated by the dashed line (Figure 3) seems to result in no major change here.

**Figure 4:**
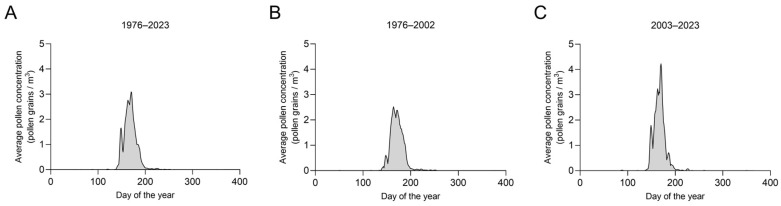
Average daily pollen concentration (grains per m^−3^) of *Ailanthus*. (**A**) over the whole observation period and the two different collection sites, (**B**) representing the years 1976–2002 (without 1996), and (**C**) 2003–2023. Curve smoothed by 6th order polynomial rolling average over 9 values.

**Figure 5:**
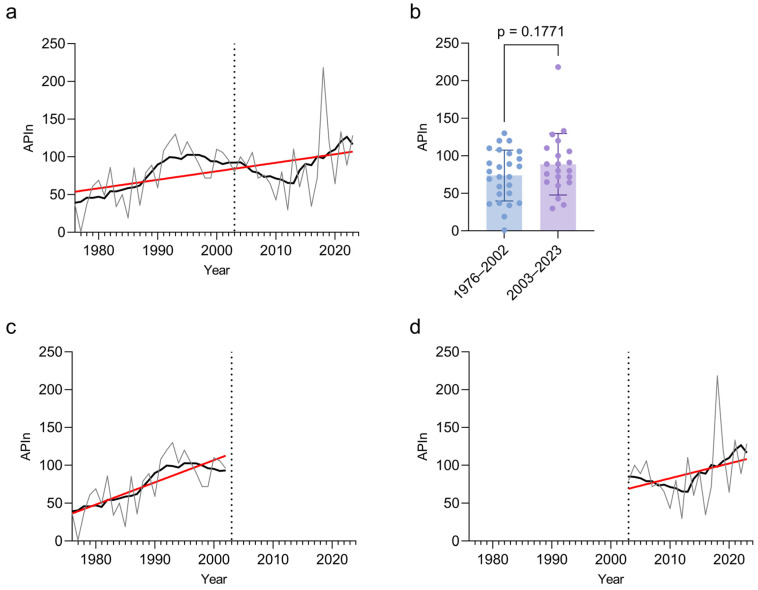
Annual pollen integral (APIn) of *Ailanthus* over time. (**a**) Analysis of *Ailanthus* APIn over the whole observation period. Thin gray line: raw APIn; black line: smoothed curve by 0th order polynomial rolling average over 8 values; red line: linear regression of APIn (R^2^ = 0.181, Slope = 1.129, Intercept = −2178, F-test for slope deviation from 0: *p* = 0.0029). (**b**) Comparison of the individual APIn of the two different collection periods 1976–2002 and 2003–2023 showing no significant difference between the two periods/sites. Two-tailed *t*-test. (**c**) as A, but for period 1976–2002 (without 1996). Linear regression of APIn (R^2^ = 0.4774, Slope = 2.938, Intercept = −5770, F-test for slope deviation from 0: *p* < 0.0001). (**d**) as A, but for period 2003–2023. Linear regression of APIn (R^2^ = 0.088, Slope = 1.958, Intercept = −3852, F-test for slope deviation from 0: *p* = 0.1916). The dotted line indicates the year 2002/2003 where the pollen collection point was moved between the two sites.

The average pollen concentrations curve shows a change, when comparing both time intervals (1976–2002 and 2003–2023) with the average of the whole dataset (1976–2023; Figure 4): the average pollen concentrations increased in the more recent time interval showing a higher first peak and a higher total peak of the season. The APIn increased over time for both the whole time period (Figure 5a), as well as when analyzing the time periods separately (Figure 5c,d). There was no statistically significant difference in the APIn in the dataset from 1976 to 2002 compared to that from 2003 to 2023 (Figure 5b).

### 2.2. The Relationship of Weather and the Ailanthus Pollen Season

**Figure 6:**
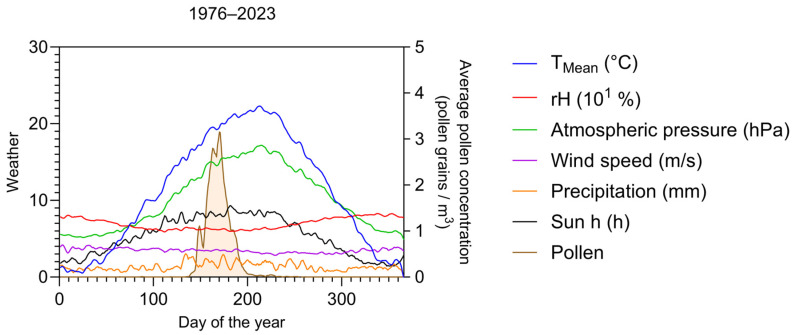
Climatic diagram for the years 1976–2023 (excluding 1996), showing on the left y-axis averages of mean temperature (T_mean_), atmospheric pressure (hPa), wind speed (m/s), relative humidity (rH), precipitation (mm), number of sun hours, overlaid with average pollen concentration (grains per m^−3^) on the right y-axis. Curves smoothed by 6th order polynomial rolling average over 9 values.

The weather parameters are shown throughout the day of the year with the average pollen concentration for the whole data series (Figure 6). Whereas there is no obvious influence of most parameters (like atmospheric pressure), T_mean_ shows at a certain point around DOY 140 a common increase with the average pollen concentration. This graph is based on the average of the nearly five-decade-long data series.

**Figure 7:**
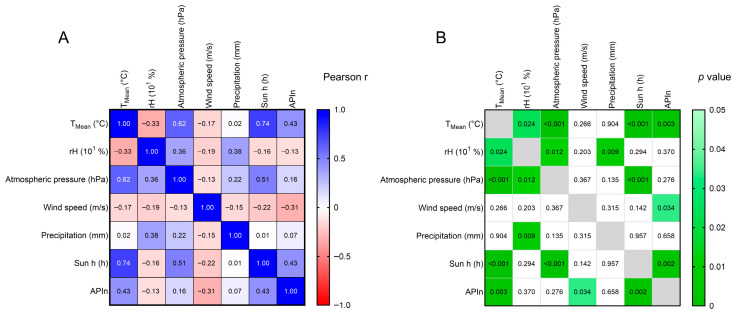
Correlation analysis of *Ailanthus* pollen with climatic parameters. (**A**) Heatmap displaying Pearson correlation coefficients r (*r* = 1: 100% positive correlation, *r* = 0: no correlation, *r* = −1: 100% negative correlation) for averaged parameters per year over the whole observation period 1976–2023 (excluding 1996); daily mean temperature (T_mean_), daily relative humidity (rH), daily atmospheric pressure (hPa), daily wind speed (m/s), daily precipitation, daily number of sun hours and daily pollen concentrations (APIn). Blue = positive correlation, white = no correlation, red = negative correlation. Values in the cells are the numerical person r coefficients. (**B**) Heatmap displaying the *p*-values of the Pearson correlation (white = *p* > 0.05, shades of green *p* < 0.05). Values in the cells are the numerical *p*-values.

Diving deeper into the comparison of weather and pollen data, more insights appear. Pearson correlation analysis showed that the APIn is correlated most with sun h and T_mean_ (Pearson correlation coefficient of 0.43 in both cases; see Figure 7), while wind speed is negatively correlated with the APIn (−0.31; Figure 7).

## 3. Discussion

### 3.1. Climate Change Increases Pollen Levels

This is the first *Ailanthus* pollen dataset that comprises more than four decades and allows first insights over a long period of time. The following results could be gained: (i) the APIn of *Ailanthus* increases with time, (ii) the season starts and peaks are earlier and (iii) the APIn is correlated with increasing mean temperatures and sunshine hours. These results are in line with many other results on plant flowering regarding global climate change [33]. The average annual air temperature increased in Austria by 1.8 °C already ([34]; observed for the last decades in the lowlands). This is 20% higher compared to other global land areas [34]. For cities, especially for a metropolis like Vienna, the urban heat island effect is an additional key factor [35]: this effect describes the faster and earlier heating within a city due to higher temperatures at night. Therefore, urban trees in a metropolis are subjected to overall higher temperatures. Other invasive plant species such as late-flowering *Artemisia* neophytes also seem to benefit from higher temperatures in Vienna and contribute to a second pollen peak in autumn, as has been demonstrated in recent years [36]. Temperatures and rainfall are key factors for the changing pollen production due to climate change [18]. Therefore, it is likely that the *Ailanthus* pollen season increased in intensity due to more favorable climate conditions. Pollen production may be elevated and a larger number of plants release pollen in areas that are not controlled by governmental authorities.

Different insects are known as pollinators of *Ailanthus*. The most frequent flower visitors are bees, flies and ants [19]. Insect-pollinated plants have a higher chance of being pollinated during sunny periods, which promotes pollen release. As pollinators such as honeybees prefer visiting flowers during sunshine, it is not surprising that also sunshine hours correlate significantly with the average pollen concentrations in the air. However, microscopical analysis does not reveal information on the fertility of these pollen grains. In fact, insect-pollinated plants were previously shown to react to climate change-induced abiotic stress in a number of ways, including pollen sterility, reduced pollen viability and reduced pollen tube length [37,38].

### 3.2. Local and Regional Distribution of Ailanthus in and Around Vienna

The increase in the annual pollen integral was observed, suggesting an expansion of this species in the urban area of Vienna. This may be because *Ailanthus* is well-adapted to the stressors in cities (see Introduction), as well as to the fact that it further expands and is not controlled in regions where it is already established [29]. Another competitive advantage is the reproductivity and vegetative propagation of the tree. Plants can produce seeds already in the third vegetation period, seed production is high, dispersal by wind and water is efficient, and clonal root suckers are formed after cutting of the parent plant [1,39]. Therefore, it can easily spread further from invaded areas.

Details of the spread of *Ailanthus* trees in Vienna are sparse since studies and data sources are either incomplete or unpublished [40]. The highest frequency of *Ailanthus* in Vienna is on industrial brownfields and inner cities’ courtyards, with an increased abundance on railway territory [40]. Detailed mapping of *Ailanthus* in Vienna was performed in the 1990s and focused on the hot spots in the western, southeastern and central parts of the city [40]. About 4,120 individuals were reported on 241 locations and an extremely strong increase in young individuals was recognized, suggesting that the total number of individuals was rising exponentially [40]. The result of this study is indirectly confirmed in the neobiota strategy and management plan of Vienna (Figure 1; [29]), suggesting a further spread of *Ailanthus* to the east and, in a lower extent, the northwestern parts of the city. However, the estimated distribution of trees is unknown and may be much higher in unmapped areas.

The pollen data timeline included in this study changed in 2003 from an inner-city location to a northern district of Vienna (see Material and Methods) close to the Vienna Woods, where *Ailanthus* is only appearing on occasion (Figure 1; [29]). However, also in this location, the APIn increased, which strengthens the hypothesis of an expansion in the whole Vienna area as well as more beneficial conditions for pollen production due to increasing temperatures, given the primary insect-pollinated nature of *Ailanthus*. The higher presence of pollen of this urban tree can also be retrieved from honey samples in Vienna [41]: 49 out of 50 samples contained *Ailanthus* pollen, with it being the predominant pollen type (>45%) in 15 of these samples. *Ailanthus* honey is able to harmonize diverse flavor profiles and is characterized by a fruity taste [42].

Moreover, the spread of *Ailanthus* and its invasive nature is depicted by the increased establishment of this taxon along the motorways and industrial areas outside the city and into natural habitats such as oak forests in Austria (project report for protected area management in Lower Austria; [43]). Also, the neighboring countries report propensities of the spread of *Ailanthus* in the great plain region in Hungary [44] and into the Danube flood plain forests in Slovakia [45].

### 3.3. Limitations of This Study

Every methodology has its limitations and the following points that may have had an impact on the results should be addressed. The long period and retrospective nature of the analysis require dealing with a heterogeneous data quality, especially regarding the two locations and missing data in some years. The year 1996 was excluded from the statistical analysis since data was missing within the season (see Material and Methods). Apart from that, larger amounts of missing data were mostly observed in the 1990s in autumn and winter (Figure 3), which is corresponding with the vegetation dormancy period in Austria. As the pollen release of *Ailanthus* is restricted to late spring and early summer, the missing values outside the vegetation period are not influencing the statistical approach of the study.

Pollen measurements may show local differences, especially when monitoring samplers are close to the emission source. Hence, a location change might influence the results, and the comparison of different timelines could be challenging. Here, the increase in the APIn of *Ailanthus* was observed for both time periods—before and after the site change (Figure 5c,d). Although Figure 5 shows a general drop that also continues after the site change, it is not likely that the *Ailanthus* pollen data is significantly affected by the change in location of the pollen measurements.

Despite global warming seeming to be a favorable factor for the distribution and pollen production of some plant species, it should be emphasized that not every plant profits or will profit from climate change. Pollen seasons of different taxa may extend, shorten, show variations in intensity or are not affected so far, depending on the respective plant [33]. As a thermophilic, invasive species, *Ailanthus altissima* is a beneficiary of increasing temperatures in a metropolis like Vienna.

Summarizing, *Ailanthus* is an insect-pollinated tree, although pollen is also in relevant concentrations in the air. The spread of *Ailanthus* continues and is reflected in the pollen data. This tree profits from climate change and is adapted to the challenging conditions in urban environments. In the future, the increase in temperature is expected to continue and further shape the pollen seasons and the flower of urban trees like *Ailanthus.* This will also impact people allergic to pollen and may result in an increase in sensitizations. Long-term datasets covering decades are essential to assess this development, and pollen monitoring must be continued to track ongoing changes.

## 4. Materials and Methods

### 4.1. Area Characteristics

The device for aerobiological monitoring was a Burkard Manufacturing Co., Ltd. pollen trap of the Hirst design [46] that is situated in Vienna (Austria). However, the Vienna pollen trap moved within the observation period. It was situated on the rooftop of the ENT department of the General Hospital of Vienna (AKH) (Latitude 48.21972; longitude 16.34833; height above sea level 200 m; height above ground level 20 m) from 1976 to 2002 (location A, Figure 1). The location is situated in a fully urbanized area and close to the Vienna city center. The vegetation of the surrounding area is characterized by typical city trees and hedge plants. There are no larger green spaces in the vicinity of the location except for smaller parks and courtyards. In 2003, the pollen monitoring station was moved to the rooftop of the main building of the GeoSphere Austria (Latitude 48.24889; longitude 16.35611; height above sea level 209 m; height above ground level 9 m; location B, Figure 1). This location is situated in a suburban area of Vienna without directly adjacent buildings, but rather, gardens and individual buildings nearby. The vegetation of the surrounding area consists mainly of trees, shrubs and herbaceous plants. Moreover, the location is influenced by the Vienna Woods, which are composed of deciduous forest vegetation typical of Central Europe. The two locations are approximately 3.3 km apart (Figure 1). Although the locations show a slightly different land use setting, the calculated degree of urbanization is comparable [47] and both sites show similar preconditions regarding the occurrence of *Ailanthus altissima*.

### 4.2. Aerobiological Monitoring, Sampling and Preparation

Pollen data was recorded as daily airborne pollen concentrations. The volumetric pollen trap transfers airborne particles to a plastic tape that is attached to a rotating drum by sucking in ambient air at a rate of 10 L/min through a 2 mm × 14 mm orifice. The tape is exchanged once or twice per week for sampling and is divided into sections that correspond to 24 h periods. An embedding solution of distilled water, glycerol, phenol, Mowiol^®^ and basic fuchsine is used to mount each 24 h segment of the tape between a light microscopy object slide and a cover slip. The samples were analyzed with an Olympus BH2 light microscope (40× magnification). Particles were counted in three longitudinal transects to express airborne pollen as mean daily pollen concentration per cubic meter of air following the minimum recommendations of the aerobiological community [48] and the European Standard [49]. These data quality parameters were applied by Siegfried Jäger from the very beginning as he was the first to evaluate pollen data of Vienna and before those guidelines were published. Pollen data was assessed by two analysts at the Medical University of Vienna: Siegfried Jäger (for 1976–2011) and Maximilian Bastl (for 2011–2023).

A percentage definition (from 2.5 to 97.5%) was applied to define the seasons. Such season definitions are recommended for retrospective purposes [50]. This means that the start day is the day on which 2.5% of the APIn (following [48]) is registered and the end date is the day on which 97.5% of the APIn is recorded [51,52]. The annual pollen integral (APIn) was calculated for the whole year over all investigated years, which were selected based on the most frequent occurrence of *Ailanthus* pollen in the air. All pollen season descriptors per individual season are given in Supplementary Table S3. The averaged pollen season descriptors for the analyzed time period are given in Table 2.

### 4.3. Weather Data

Weather data was requested from the “Hohe Warte”, GeoSphere Austria in Vienna. It was monitored and provided by the GeoSphere Austria itself. The contained parameters are the mean temperature (T_mean_), relative humidity (rH), atmospheric pressure, wind speed, precipitation (prec.) and sun hours (sun h). For part of the time period, the pollen monitoring was performed on the “Hohe Warte” location itself (2003–2023; see above).

### 4.4. Data Analysis

The AeRobiology [53] library (v2.0.1) with R (v4.4.0, R Foundation for Statistical Computing, Vienna, Austria) and RStudio (v2024.09.0+375) [54] was used for exploratory data analysis, quality control and trend analysis.

The quality of the *Ailanthus altissima* historical data corpus was evaluated through the *quality_control* function, which examines three primary metrics: (i) the extent of data completeness throughout the core pollen season, (ii) the availability of data at the start, peak and end dates of the season, and (iii) the proportion of missing data within the main pollen period. In a first step, the total missing values for each year were evaluated (Supplementary Table S1). Since more than 50% of the values were missing in the year 1996, we decided to exclude this year for subsequent analyses. Afterwards, the annual pollen concentration records were analyzed using a risk classification system, with special emphasis on the pollination period.

The quality control algorithm returned TRUE/FALSE values for meeting specified criteria for the entire year (‘Complete’), the key seasonal parameters (‘Start’, ‘Peak’, ‘End’), and the main pollen season itself (‘Comp.MPS’). This assessment was based on a 2-day sliding window approach and a tolerance of a maximum of 20% missing values within the main pollen season, yielding a risk score between 0 (no risk) and 5 (high risk) (Supplementary Table S2). This rating reflects the potential consequences of incorporating the dataset into subsequent analyses, considering gaps in data coverage across the full pollen season and near pivotal dates. The analysis indicated a low risk for the year 1997 due to missing values in the beginning of the pollen season. However, since the main season, as well as the peak and the end was recorded for the year 1997, it was included for subsequent analyses. All other years, apart from 1996, did not show a significant risk.

Main pollen season descriptors (start date, peak date, end date and annual pollen integral) were estimated using the *calculate_ps* function from the AeRobiology package in R. The pollen season was defined using the percentage method, with a threshold of 95% of the annual total pollen. To address missing data, gaps were interpolated dynamically via a moving mean method: missing values were replaced by the mean of daily pollen concentrations within a window size determined by multiplying the gap length by a factor of 2 [55]. Years with large data gaps during the *Ailanthus* pollen season were omitted (year 1996). Years with data gaps outside the *Ailanthus* pollen season were included as they do not affect the analysis negatively, e.g., pollen concentrations are very low during vegetation dormancy (autumn- and wintertime) and *Ailanthus* pollen is rarely found outside its main flowering time. Temporal trends in the seasonal indices were analyzed using the *analyse_trend* and *plot_trend* functions (AeRobiology). Simple linear regression was applied to assess changes over time, with regression slopes and associated *p*-values reported to quantify trend significance. Smoothed trends (with 95% confidence intervals) and linear regression fits were visualized to evaluate long-term patterns.

Graphing and statistical analysis of *Ailanthus* pollen and weather data were performed using GraphPad Prism (Version 10.5). Smoothing functions, regression models and statistical tests are indicated in the respective figure legends. Correlation of weather parameters (mean temperature, relative humidity, atmospheric pressure, precipitation, wind speed and sun hours) were connected to the observed APIn, average yearly weather data and APIn for each year were analyzed by Pearson correlation analysis (Figure 7). Single individual missing data points for days adjacent to days with positive pollen numbers (i.e., non-zero) were interpolated by averaging. The year 1996 was excluded from the analysis as too many data points during the pollen season were missing. The data for the intervals 1976–2002 and 2003–2023 were analyzed both separately and together (whole observation period).

## Figures and Tables

**Table 1:** *Ailanthus altissima* pollen season descriptors for Vienna. Data was averaged over the 1976–2023 period (excluding 1996).

Pollen Season Descriptor	Vienna (n = 47)	
	Mean	SD ^+^
Annual pollen integral, grains m^−3^	82	41
Season start date (2.5%), DOY *	158	11
Season end date (97.5%), DOY	194	16
Season length, n. of days	37	17
Peak value, grains m^−3^	17	14
Peak date, DOY	167	10
Length of pre-peak period, days	11	7
Pollen integral of pre-peak period, grains m^−3^	44	28
Length of post-peak period, days	27	16
Pollen integral of post-peak period, grains m^−3^	38	23

* Day of Year. ^+^ Standard deviation.

**Table 2:** Trend analysis for pollen descriptors of *Ailanthus altissima* from 1976 to 2023 (excluding 1996).

Variable	Coefficient	*p*-Value
Season start date (2.5%), DOY *	−0.3373036	0.001348889
Peak date, DOY	−0.2738010	0.005149643
Season end date (97.5%), DOY	−0.1619008	0.325559800
Annual pollen integral, grains m^−3^	1.0797401	0.004314875

* Day of Year.

## Data Availability

All relevant data are presented in this work. The raw pollen data from Vienna are available for scientific purposes on request and agreement with the corresponding author. The raw weather data must be requested from the GeoSphere Austria itself.

## Supplementary Materials

The following supporting information can be downloaded at: https://www.mdpi.com/article/10.3390/plants14243823/s1, Table S1: Missing values in *Ailanthus altissima* historical dataset; Table S2: Quality control risk table of *Ailanthus altissima* dataset; Table S3: *Ailanthus altissima* pollen season descriptors by individual season.

## Abbreviations

The following abbreviations are used in this manuscript:

APIn	Annual pollen integral
Prec.	Precipitation
rH	Relative humidity
Sun h	Sun hours
T_mean_	Mean temperature
DOY	Day of year
