# Supplementary material for: The Impact of Climate Change on the Urban Tree Ailanthus altissima: Insights from More than Four Decades of Pollen Data in Vienna (Austria)
Source: Plants (Basel). 2025 Dec 16;14(24):3823. doi: 10.3390/plants14243823 (PMC12736547; doi:10.3390/plants14243823)
Supplement: Supplementary file 1 [file plants-14-03823-s001.zip › plants-3947550-supplementary/SuppTableS1_missing_data_V3.pdf]

Supplementary Table S1. Missing values in *Ailanthus altissima* historical data set.

Year	Missing values (n)	Missing values (%)
1976	0	0
1977	0	0
1978	0	0
1979	0	0
1980	0	0
1981	0	0
1982	0	0
1983	0	0
1984	0	0
1985	0	0
1986	0	0
1987	0	0
1988	0	0
1989	0	0
1990	0	0
1991	103	28.93
1992	101	28.37
1993	113	31.74
1994	93	26.12
1995	138	38.76
1996	185	51.97
1997	147	41.29
1998	98	27.53
1999	137	38.48
2000	82	23.03
2001	72	20.22
2002	14	3.93
2003	0	0
2004	0	0
2005	0	0
2006	0	0
2007	0	0
2008	0	0
2009	1	0.28
2010	9	2.53
2011	13	3.65
2012	1	0.28
2013	2	0.56
2014	0	0
2015	0	0
2016	2	0.56
2017	0	0
2018	0	0
2019	0	0
2020	4	1.12
2021	2	0.56
2022	9	2.53
2023	0	0
