# Supplementary material for: The Impact of Climate Change on the Urban Tree Ailanthus altissima: Insights from More than Four Decades of Pollen Data in Vienna (Austria)
Source: Plants (Basel). 2025 Dec 16;14(24):3823. doi: 10.3390/plants14243823 (PMC12736547; doi:10.3390/plants14243823)
Supplement: Supplementary file 1 [file plants-14-03823-s001.zip › plants-3947550-supplementary/SuppTableS2_risk-table_revision.pdf]

Supplementary Table S2. Quality control risk table of *Ailanthus altissima* data set.

Season	Complete	Start	Peak	End	Comp.MPS	Risk
1976	TRUE	TRUE	TRUE	TRUE	TRUE	0
1977	TRUE	TRUE	TRUE	TRUE	TRUE	0
1978	TRUE	TRUE	TRUE	TRUE	TRUE	0
1979	TRUE	TRUE	TRUE	TRUE	TRUE	0
1980	TRUE	TRUE	TRUE	TRUE	TRUE	0
1981	TRUE	TRUE	TRUE	TRUE	TRUE	0
1982	TRUE	TRUE	TRUE	TRUE	TRUE	0
1983	TRUE	TRUE	TRUE	TRUE	TRUE	0
1984	TRUE	TRUE	TRUE	TRUE	TRUE	0
1985	TRUE	TRUE	TRUE	TRUE	TRUE	0
1986	TRUE	TRUE	TRUE	TRUE	TRUE	0
1987	TRUE	TRUE	TRUE	TRUE	TRUE	0
1988	TRUE	TRUE	TRUE	TRUE	TRUE	0
1989	TRUE	TRUE	TRUE	TRUE	TRUE	0
1990	TRUE	TRUE	TRUE	TRUE	TRUE	0
1991	TRUE	TRUE	TRUE	TRUE	TRUE	0
1992	TRUE	TRUE	TRUE	TRUE	TRUE	0
1993	TRUE	TRUE	TRUE	TRUE	TRUE	0
1994	TRUE	TRUE	TRUE	TRUE	TRUE	0
1995	TRUE	TRUE	TRUE	TRUE	TRUE	0
1997	TRUE	**FALSE**	TRUE	TRUE	TRUE	**1**
1998	TRUE	TRUE	TRUE	TRUE	TRUE	0
1999	TRUE	TRUE	TRUE	TRUE	TRUE	0
2000	TRUE	TRUE	TRUE	TRUE	TRUE	0
2001	TRUE	TRUE	TRUE	TRUE	TRUE	0
2002	TRUE	TRUE	TRUE	TRUE	TRUE	0
2003	TRUE	TRUE	TRUE	TRUE	TRUE	0
2004	TRUE	TRUE	TRUE	TRUE	TRUE	0
2005	TRUE	TRUE	TRUE	TRUE	TRUE	0
2006	TRUE	TRUE	TRUE	TRUE	TRUE	0
2007	TRUE	TRUE	TRUE	TRUE	TRUE	0
2008	TRUE	TRUE	TRUE	TRUE	TRUE	0
2009	TRUE	TRUE	TRUE	TRUE	TRUE	0
2010	TRUE	TRUE	TRUE	TRUE	TRUE	0
2011	TRUE	TRUE	TRUE	TRUE	TRUE	0
2012	TRUE	TRUE	TRUE	TRUE	TRUE	0
2013	TRUE	TRUE	TRUE	TRUE	TRUE	0
2014	TRUE	TRUE	TRUE	TRUE	TRUE	0
2015	TRUE	TRUE	TRUE	TRUE	TRUE	0
2016	TRUE	TRUE	TRUE	TRUE	TRUE	0
2017	TRUE	TRUE	TRUE	TRUE	TRUE	0
2018	TRUE	TRUE	TRUE	TRUE	TRUE	0
2019	TRUE	TRUE	TRUE	TRUE	TRUE	0
2020	TRUE	TRUE	TRUE	TRUE	TRUE	0
2021	TRUE	TRUE	TRUE	TRUE	TRUE	0
2022	TRUE	TRUE	TRUE	TRUE	TRUE	0
2023	TRUE	TRUE	TRUE	TRUE	TRUE	0

Complete: Logical check indicating whether a main pollen season could be calculated (TRUE) or not (FALSE).
Start, Peak, End: Logical checks indicating whether the key seasonal parameters are based on robust, non-interpolated data (TRUE) or are potentially biased by missing data on or near the date (FALSE).

Comp.MPS: Logical check indicating whether the percentage of missing data within the main pollen season is at or below the acceptable threshold of 20% (TRUE) or exceeds it (FALSE).
Risk: Risk score (0-5) based on the distribution of missing data throughout the entire year, where 0 indicates no risk and 5 indicates high risk.
