# Supplementary material for: The Impact of Climate Change on the Urban Tree Ailanthus altissima: Insights from More than Four Decades of Pollen Data in Vienna (Austria)
Source: Plants (Basel). 2025 Dec 16;14(24):3823. doi: 10.3390/plants14243823 (PMC12736547; doi:10.3390/plants14243823)
Supplement: Supplementary file 1 [file plants-14-03823-s001.zip › plants-3947550-supplementary/SuppTableS3_pollen_season_data_V2.pdf]

Supplementary Table S3: *Ailanthus altissima* pollen season descriptors by individual season.

Season	St.dt	St.jd	En.dt	En.jd	Ln.ps	Sm.tt	Sm.ps	Pk.val	Pk.dt	Pk.jd	Ln.prpk	Sm.prpk	Ln.pspk	Sm.pspk	Daysth
1976	1976-06-30	182	1976-08-02	215	34	37	37	8	1976-06-30	182	1	8	33	29	0
1977	1977-06-27	178	1977-06-27	178	1	1	1	1	1977-06-27	178	1	1	0	1	0
1978	1978-06-23	174	1978-08-08	220	47	37	37	8	1978-07-03	184	11	20	36	17	0
1979	1979-06-06	157	1979-06-28	179	23	61	59	9	1979-06-11	162	6	19	17	40	0
1980	1980-06-27	179	1980-07-22	204	26	69	67	10	1980-07-01	183	5	19	21	48	0
1981	1981-06-09	160	1981-06-28	179	20	49	47	10	1981-06-13	164	5	28	15	19	0
1982	1982-06-09	160	1982-07-01	182	23	86	84	14	1982-06-23	174	15	61	8	23	0
1983	1983-06-04	155	1983-07-08	189	35	34	34	7	1983-06-12	163	9	23	26	11	0
1984	1984-06-23	175	1984-07-11	193	19	50	49	5	1984-06-28	180	6	18	13	31	0
1985	1985-06-12	163	1985-07-14	195	33	19	19	2	1985-06-12	163	1	2	32	17	0
1986	1986-06-06	157	1986-07-07	188	32	85	82	11	1986-06-19	170	14	55	18	27	0
1987	1987-06-12	163	1987-07-11	192	30	36	36	8	1987-06-30	181	19	19	11	17	0
1988	1988-06-04	156	1988-07-05	187	32	79	78	11	1988-06-22	174	19	42	13	36	0
1989	1989-06-09	160	1989-08-29	241	82	89	89	26	1989-06-14	165	6	44	76	45	0
1990	1990-06-12	163	1990-06-28	179	17	59	57	14	1990-06-19	170	8	31	9	26	0
1991	1991-06-26	177	1991-07-10	191	15	108	104	22	1991-07-02	183	7	58	8	46	0
1992	1992-06-05	157	1992-08-10	223	67	120	117	18	1992-06-21	173	17	64	50	53	0
1993	1993-05-25	145	1993-07-29	210	66	132	128	22	1993-06-10	161	17	81	49	47	0
1994	1994-06-09	160	1994-07-15	196	37	103	100	22	1994-06-19	170	11	39	26	61	0

Season	St.dt	St.jd	En.dt	En.jd	Ln.ps	Sm.tt	Sm.ps	Pk.val	Pk.dt	Pk.jd	Ln.prpk	Sm.prpk	Ln.pspk	Sm.pspk	Daysth
1995	1995-06-09	160	1995-08-07	219	60	122	117	22	1995-06-21	172	13	80	47	37	0
1996	1996-03-04	64	1996-07-26	208	145	4	3	1	1996-03-04	64	1	1	144	2	0
1997	1997-06-04	155	1997-07-20	201	47	137	131	10	1997-06-28	179	25	98	22	33	0
1998	1998-06-05	156	1998-06-25	176	21	72	71	17	1998-06-10	161	6	51	15	20	0
1999	1999-05-21	141	1999-07-14	195	55	72	71	16	1999-06-08	159	19	39	36	32	0
2000	2000-05-23	144	2000-06-29	181	38	110	106	38	2000-05-27	148	5	44	33	62	0
2001	2001-06-02	153	2001-08-21	233	81	106	102	15	2001-06-14	165	13	35	68	67	0
2002	2002-05-22	142	2002-07-17	198	57	96	94	14	2002-06-05	156	15	36	42	58	0
2003	2003-06-04	155	2003-06-29	180	26	80	78	14	2003-06-08	159	5	42	21	36	0
2004	2004-06-17	169	2004-08-13	226	58	100	98	42	2004-07-04	186	18	91	40	7	0
2005	2005-06-14	165	2005-07-16	197	33	89	85	22	2005-06-20	171	7	39	26	46	0
2006	2006-06-17	168	2006-07-06	187	20	106	102	25	2006-06-25	176	9	87	11	15	0
2007	2007-05-26	146	2007-07-19	200	55	72	70	8	2007-05-27	147	2	10	53	60	0
2008	2008-05-28	149	2008-07-06	188	40	76	74	8	2008-06-07	159	11	17	29	57	0
2009	2009-05-23	143	2009-07-03	184	42	65	63	16	2009-06-06	157	15	24	27	39	0
2010	2010-06-12	163	2010-07-14	195	33	43	41	5	2010-06-27	178	16	26	17	15	0
2011	2011-05-31	151	2011-06-28	179	29	80	78	19	2011-06-06	157	7	33	22	45	0
2012	2012-05-25	146	2012-06-19	171	26	27	27	6	2012-06-16	168	23	24	3	3	0
2013	2013-05-19	139	2013-07-16	197	59	114	111	27	2013-06-20	171	33	95	26	16	0
2014	2014-06-07	158	2014-06-28	179	22	62	60	14	2014-06-12	163	6	49	16	11	0
2015	2015-06-02	153	2015-07-20	201	49	92	88	21	2015-06-12	163	11	41	38	47	0

Season	St.dt	St.jd	En.dt	En.jd	Ln.ps	Sm.tt	Sm.ps	Pk.val	Pk.dt	Pk.jd	Ln.prpk	Sm.prpk	Ln.pspk	Sm.pspk	Daysth
2016	2016-06-02	154	2016-07-10	192	39	34	34	5	2016-06-17	169	16	22	23	12	0
2017	2017-06-03	154	2017-07-05	186	33	74	72	13	2017-06-09	160	7	32	26	40	0
2018	2018-05-26	146	2018-07-12	193	48	220	212	65	2018-05-29	149	4	98	44	114	0
2019	2019-06-10	161	2019-06-27	178	18	120	119	33	2019-06-12	163	3	63	15	56	0
2020	2020-06-06	158	2020-07-13	195	38	61	59	8	2020-06-13	165	8	21	30	38	0
2021	2021-06-16	167	2021-07-12	193	27	133	130	37	2021-06-18	169	3	54	24	76	0
2022	2022-05-31	151	2022-07-14	195	45	92	90	12	2022-06-15	166	16	55	29	35	0
2023	2023-06-14	165	2023-07-16	197	33	128	123	21	2023-06-20	171	7	61	26	62	0

St.dt: start date (date)
St.jd: start date (day of the year)
En.dt: end date (date)
En.jd: end date (day of the year)
Ln.ps: length of the season
Sm.tt: total sum
Sm.ps: pollen integral
Pk. Val: peak value
Pk.dt: peak date (date)
Pk.jd: peak date (day of the year)
Ln.prpk: length of the pre peak period
Sm.prpk: pollen integral of the pre peak period
Ln.pspk: length of the post peak period
Sm.pspk: pollen integral of the post peak period
Daysth: number of days with more than 100 pollen grains
